# Long-Term Treatment with Bortezomib Induces Specific Methylation Changes in Differentiated Neuronal Cells

**DOI:** 10.3390/cancers14143402

**Published:** 2022-07-13

**Authors:** Karolina Łuczkowska, Olga Taryma-Leśniak, Jan Bińkowski, Katarzyna E. Sokołowska, Dominik Strapagiel, Justyna Jarczak, Edyta Paczkowska, Bogusław Machaliński, Tomasz K. Wojdacz

**Affiliations:** 1 Department of General Pathology, Pomeranian Medical University in Szczecin, 70-115 Szczecin, Poland; karolina.luczkowska@pum.edu.pl (K.Ł.); edyta.paczkowska@pum.edu.pl (E.P.); boguslaw.machalinski@pum.edu.pl (B.M.); 2 Independent Clinical Epigenetics Laboratory, Pomeranian Medical University in Szczecin, 71-252 Szczecin, Poland; olga.taryma-lesniak@pum.edu.pl (O.T.-L.); jan.binkowski@pum.edu.pl (J.B.); katarzyna.sokolowska@pum.edu.pl (K.E.S.); 3 Biobank Lab, Department of Molecular Biophysics, Faculty of Biology and Environmental Protection, University of Lodz, 90-235 Lodz, Poland; dominik.strapagiel@biol.uni.lodz.pl (D.S.); j.jarczak@nencki.edu.pl (J.J.); 4 Laboratory of Molecular Basis of Behavior, Nencki Institute of Experimental Biology, Polish Academy of Science, 02-093 Warsaw, Poland; 5 Department of Biomedicine, Aarhus University, Hoegh-Guldbergsgade 10, 8000 Aarhus, Denmark

**Keywords:** multiple myeloma, peripheral neuropathy, bortezomib, LUHMES, methylation, epigenetics

## Abstract

**Simple Summary:**

We exposed LUHMES cells, differentiated into mature neurons, to bortezomib (BTZ) in two treatment cycles and analyzed the methylomes of these cells after each cycle, controlling the analysis for the methylation changes potentially induced by the long-term culture. Our results show that BTZ induces methylation changes that may affect cell morphogenesis, neurogenesis, and neurotransmission. These changes are specifically enriched within transcription factor binding sites of EBF, PAX, DLX, LHX, and HNF family members, which have been shown to regulate neurogenesis and neuronal differentiation. We further show that the observed methylation changes are not present in the SH-SY5Y cells that we used to study mechanisms of development of BTZ resistance. Altogether, our results show that BTZ treatment induces very specific changes in the methylomes of neuronal cells.

**Abstract:**

Bortezomib (BTZ) is proteasome inhibitor, effectively used in the treatment of multiple myeloma, but frequently discontinued due to peripheral neuropathy, which develops in patients after consecutive treatment cycles. The molecular mechanisms affected by BTZ in neuronal cells, which result in neuropathy, remain unknown. However, BTZ is unlikely to lead to permanent morphological nerve damage, because neuropathy reverses after discontinuation of treatment, and nerve cells have very limited renewal capacity. We have previously shown that BTZ induces methylation changes in SH-SY5Y cells, which take part in the development of treatment resistance. Here, we hypothesized that BTZ affects the methylomes of mature neurons, and these changes are associated with BTZ neurotoxicity. Thus, we studied methylomes of neuronal cells, differentiated from the LUHMES cell line, after cycles of treatment with BTZ. Our results show that BTZ induces specific methylation changes in mature neurons, which are not present in SH-SY5Y cells after BTZ treatment. These changes appear to affect genes involved in morphogenesis, neurogenesis, and neurotransmission. Furthermore, identified methylation changes are significantly enriched within binding sites of transcription factors previously linked to neuron physiology, including EBF, PAX, DLX, LHX, and HNF family members. Altogether, our results indicate that methylation changes are likely to be involved in BTZ neurotoxicity.

## 1. Introduction

Bortezomib (BTZ) is a first-line chemotherapeutic agent used for the treatment of multiple myeloma (MM) [1] and mantle cell lymphoma [2]. Mechanistically, BTZ reversibly inhibits 26S proteasome and interacts with the nuclear factor kappa B (NFκB) signaling pathway, inducing the inhibition of anti-apoptotic factors and, consequently, the activation of apoptosis [3,4,5]. One of the major side effects of BTZ treatment, which frequently leads to the discontinuation of chemotherapy, is neurotoxicity; this results in peripheral neuropathy [6], observed in almost 40% of patients with MM [7]. Oxidative stress [8,9], peripheral nerve inflammation [10,11], and axonal degeneration [12] have been shown to be associated with BTZ neurotoxicity, but it is unlikely that BTZ induces permanent nerve damage, because peripheral neuropathy reverses in the majority of patients after treatment modification or discontinuation [7].

We have previously shown that BTZ induces genome-wide methylation changes in human neuroblastoma SH-SY5Y cells treated with this compound, and these changes are likely to play a significant role in the development of resistance to BTZ [13]. With clear evidence of the activity of BTZ towards DNA methylation in neuroblastoma cells, we hypothesized in this study that BTZ may also affect the methylomes of mature neurons. To test this hypothesis, we treated neuronal cells, differentiated from the Lund human mesencephalic (dLUHMES) cell line, with two cycles of four low doses of BTZ. Then, to identify methylation changes attributed to the BTZ treatment, we compared the methylomes of the cells harvested after each cycle, and controlled this analysis for methylation changes that were potentially induced by the cell culture. Our data show that repetitive treatment with BTZ induces methylation changes in neuronal cells, which was not observed in other cell types treated with this compound. These changes are involved in the physiological processes associated with cell morphogenesis, neurogenesis, and, most important in the context of mature neurons, with neurotransmission; this includes processes such as the regulation of membrane potential, synaptic signaling, ion transport, and the regulation of cation channel activity. Interestingly, identified methylation changes were enriched in the binding sites of transcription factors of three families, including EBF, PAX, DLX, LHX, and HNF, which have been shown to play a key role in neurogenesis and neuronal differentiation. Additionally, we show that BTZ-induced methylation changes may affect the chromatin structure of treated cells.

## 2. Materials and Methods

### 2.1. Cell Line Maintenance and Differentiation

There is no straightforward model to study the molecular effects of treatment in neurons. Here, we used Lund human mesencephalic (LUHMES) cells, originating from healthy human embryonic mesencephalic tissue, which display favorable characteristics for neurotoxicity screening. These can be rapidly differentiated into homogeneous neurons that exhibit high-level expression of neuronal marker genes, and higher sensitivity to known neurotoxicants, when compared to SH-SY5Y neuroblastoma cells, and neural stem cells (NSC) from the human fetal brain [14]. Additionally, these cells have been shown to be suitable for long-term culture [15]. Before differentiation, we cultured LUHMES cells according to the procedures described by Scholz et al. [16]. Briefly, the LUHMES were incubated in T-75 flasks coated with fibronectin (1 mg/mL) (Sigma-Aldrich, Darmstadt, Germany) and poly-L-ornithine (1 mg/mL) (Sigma-Aldrich, Darmstadt, Germany) in Advanced DMEM/F-12 medium (Thermo Fisher Scientific, Waltham, MA, USA), modified by the addition of N-2 supplement (100×) (Thermo Fisher Scientific, Waltham, MA, USA), L-glutamine (1 mg/mL) (Sigma-Aldrich, Darmstadt, Germany), human basic fibroblast growth factor (160 μg/mL) (bFGF, R&D Systems, Minneapolis, MN, USA), and penicillin (100 U/mL) (Sigma-Aldrich, Darmstadt, Germany) in a controlled atmosphere with a temperature of 37 °C, 5% CO_2_ concentration, and saturated humidity. The proliferation medium was replaced every two days. The culture with a sufficient number of cells was then differentiated into mature neurons according to the protocol described by Harris et al. [17]. The cell differentiation medium contained: Advanced DMEM/F-12 medium, N-2 supplement (100×), L-glutamine (1 mg/mL), cAMP (100 mM) (R&D Systems, Minneapolis, MN, USA), tetracycline (2 mg/mL) (Sigma-Aldrich, Darmstadt, Germany), and glial cell line-derived neurotrophic factor (20 μg/mL) (GDNF, R&D Systems, Minneapolis, MN, USA). The cells were incubated in these conditions for seven days and subjected to BTZ treatment.

### 2.2. BTZ Dose Determination

BTZ dose was determined experimentally to only slightly reduce the viability of cells after one cycle of treatment with BTZ (see Section 2.3: Experimental design). The viability was tested by measuring the mitochondrial membrane potential (∆ψM) (JC-1 Mitochondrial Membrane Potential Assay Kit, Cayman, Ann Arbor, MI, USA), according to the manufacturer’s protocol. The final dose of BTZ used in subsequent experiments was 0.15 nM, and at this BTZ concentration, the viability of cells decreased by only 3.76 percent points compared to non-treated controls.

### 2.3. Experimental Design

Treatment with BTZ was conducted in two cycles. In one treatment cycle, dLUHMES cells were incubated four times (on the 1st, 4th, 8th, and 11th day) with medium containing 0.15 nM of BTZ (Cell Signalling Technology, Danvers, MA, USA) for 24 h. The regimen of treatment within the cycle was designed to resemble the standard treatment of patients with MM (PMID, 15461622; PMID, 12826635). The medium was replaced with BTZ-free medium between each treatment. After the first treatment cycle, cells were cultured for another 10 days in BTZ-free medium, and then the BTZ treatment cycle was repeated. The cells treated with BTZ, as well as non-treated controls, were harvested after each cycle (referred to as “time point I” and “time point II”) for DNA extraction.

### 2.4. DNA Extraction

DNA was extracted from three separate cell incubations for each of the groups after each BTZ treatment cycle. Total DNA was extracted from 1.5 × 10^6^ dLUHMES cells using NucleoSpin TriPrep (Macherey Nagel, Düren, Germany), following the manufacturer’s instructions. The concentration and quality of obtained DNA were assessed by Qubit^®^ 2.0 Fluorometer 2.0 (Invitrogen, Waltham, MA, USA).

### 2.5. Genome-Wide Methylation Analyses

Methylation changes in both BTZ-treated and control cells were screened using the Illumina MethylationEPIC BeadChip array. This microarray allows the assessment of methylation status at more than 850,000 CpG loci. The raw data were processed with ChAMP pipeline [18] with default data processing settings, and after quality control, our dataset contained methylation data (beta values) for a total of 735,074 CpG sites.

### 2.6. Identification of Methylation Changes Attributed to BTZ Treatment

We used linear regression to compare the methylation levels at screened CpG sites between experiential time points, with the time point as exogenous and methylation levels as endogenous (response) variables. The methylation difference between the time points was expressed as a slope coefficient in the regression model. With this approach, we first identified, in control cells, CpG sites that did not change methylation status between experimental time points (FDR-corrected *p*-value > 0.05, Wald test, and absolute slope coefficient ≤ 0.025), and excluded the remaining CpGs from the analysis, as methylation levels at these CpG sites were potentially attributed to experimental conditions. Then, we identified CpG sites with stable methylation levels across the time points in BTZ-treated cells (FDR-corrected *p*-value > 0.05, Wald test, and absolute slope coefficient ≤ 0.025), and displayed the same methylation level to control cells at the first time point (FDR-corrected *p*-value > 0.05, Mann–Whitney U test). All remaining CpGs were considered as potentially induced by BTZ-treatment, and used for further analysis. Lastly, we defined CpG sites as being most affected by BTZ treatment, as the absolute value of the slope between experimental time points for CpGs was more than 0.1, which corresponds to a methylation level change of at least 10 percent points.

### 2.7. Functional Analysis

In the enrichment analysis, annotations of “Relation to UCSC CpG Island”, “UCSC RefGene Group”, and “Regulatory Feature Group” from Illumina manifest were used. The statistical significance between expected and observed frequencies of identified CpGs was tested using the chi-square goodness-of-fit test. Functional analysis of genes harboring these CpGs was performed using FUMA [19], with a background containing all genes with at least one probe available (based on Illumina annotations) in the dataset obtained after raw data processing. Transcription factor (TF) motif enrichment analysis was performed using the findMotifsGenome.pl script from HOMER [20] with the following parameters: hg19 genome, 200 bp upstream and downstream from each CpG site, and with all assessed EPIC array probes as a background. The motif enrichment was identified using cumulative hypergeometric distribution. The analysis of enrichment of BTZ-induced methylation changes in open (i.e., accessible) chromatin regions was performed using assay for transposase-accessible chromatin with sequencing (ATAC-Seq) data available for differentiated LUHMES control cells (GSE125660). The statistical significance between expected and observed frequencies of identified CpGs in open chromatin regions was tested using the chi-square goodness-of-fit test.

### 2.8. High-Dimensional Data Visualization

Data with more than three dimensions were visualized in the form of heatmaps clustered using an unsupervised Ward’s method and assuming Euclidian distance.

### 2.9. Statistical Analysis

Statistical analyses were performed in the Python 3 environment. We set the level of significance as 0.05 (alpha). Regression models were estimated using the ordinary least squares estimator.

### 2.10. Validation of Identified CpGs in Independent Experiment Data

To assess the specificity of identified methylation changes, we compared the methylation levels at the CpG sites that exhibited methylation changes after BTZ treatment in dLUHMES cells with the methylation levels observed in SH-SY5Y cells, used in our previous analysis of the influence of BTZ treatment on cell methylomes [13].

## 3. Results

### 3.1. Long-Term Culture of dLUHMES Cells Does Not Affect Methylation Status of the Vast Majority of CpG Sites Targeted by EPIC Array

Our experiment required a relatively long-term culture period of cells after differentiation into mature neurons (see Methods for details). Cell culturing conditions have been shown to induce methylation changes, especially in the case of relatively long-term treatment [21,22]; thus, in our study, we first needed to identify and exclude from the analysis CpG sites that changed methylation status due to the culturing conditions. The comparison of the methylation levels at all informative CpG sites in control cultures between two study time points (see Methods for details) showed that methylation at the vast majority of CpG sites (447,587; 60.9%) remained stable throughout our experiment (FDR-corrected *p*-value > 0.05, Wald test, and absolute slope coefficient ≤ 0.025). At the same time, the methylation level at the remaining 287,487 CpGs was different between experimental time points in the control cultures, and thus, potentially affected by the experimental conditions; these CpG sites were excluded from further analysis.

### 3.2. Globally Bortezomib Treatment Induces Hypomethylation in dLUHMES Cells

**Figure 1:**
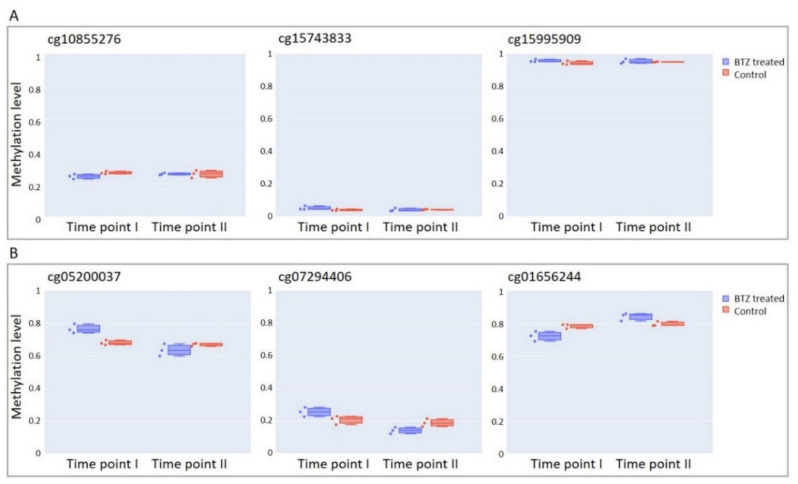
Examples of CpG sites with (**A**) stable and (**B**) changed methylation levels between experimental time points in both BTZ-treated cells and controls. Time point I: after the first cycle of BTZ treatment. Time point II: after the second cycle of BTZ treatment. The blue box indicates BTZ-treated and the orange box non-treated control cells.

We next compared the methylation levels at the CpG sites that did not show methylation changes between experimental time points in control cultures, and in cells treated one time and two times with BTZ (see Methods for details). The methylation levels at the majority of these CpG sites (367,538; 82.1%) did not change between time points in cells treated with BTZ (FDR-corrected *p*-value > 0.05, Wald test, and absolute slope coefficient ≤ 0.025), and did not show statistically significant differences (FDR-corrected *p*-value > 0.05, Mann–Whitney U test) compared to the control cells at the first time point (Figure 1A). The methylation levels at the remaining 80,049 CpGs significantly differed between experimental time points (Figure 1B), and these methylation changes are most likely attributed to the exposure to BTZ. Interestingly, the vast majority of these CpGs (49,686; 62.1%) displayed a loss of methylation between the treatments, indicating that, at the genome level, BTZ induces hypomethylation.

### 3.3. Bortezomib Treatment Induces Hypermethylation at Specific Subset of CpG Sites

**Figure 2:**
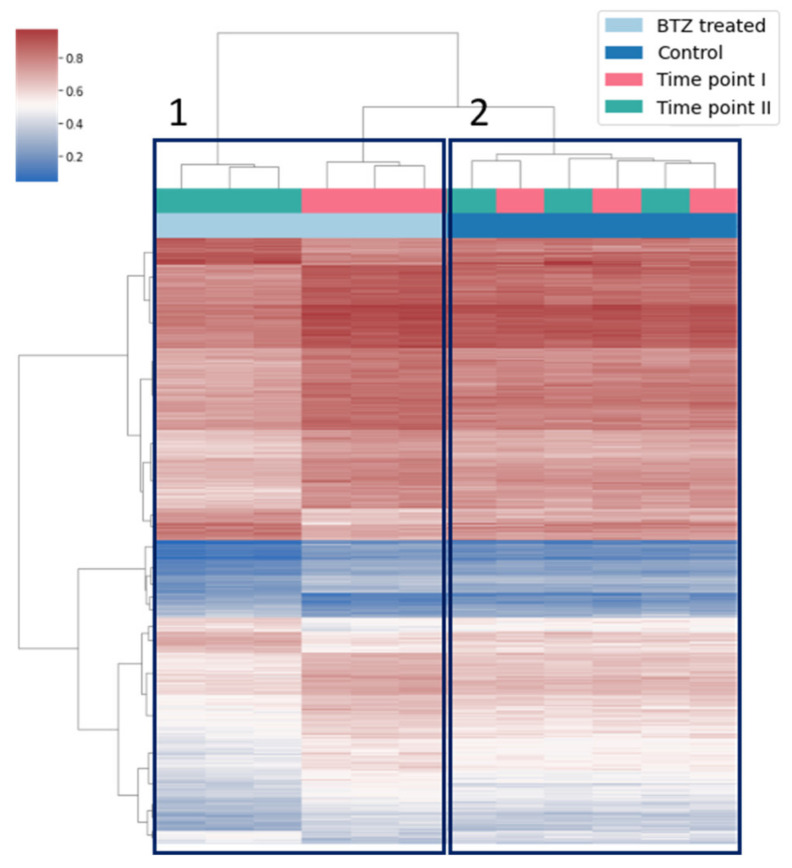
Heatmap illustrating unsupervised clustering of beta values at 687 identified CpGs in BTZ-treated and non-treated control cells at both time points. **Box 1** indicates BTZ-treated cells, and **Box 2** non-treated cells, which clustered according to treatment condition.

The subset of 80,049 CpG sites showed differences in methylation levels between the time points in BTZ-treated culture. However, only for 687 (0.8%; listed in Supplementary Table S1) of these CpG sites the absolute value of slope between experimental time points was more than 0.1, which corresponds to methylation level change of at least 10 percent points; we considered only this subset of CpG sites in further analysis. Among these CpGs, 539 gained and 148 lost methylation across the time points. Not surprisingly, the unsupervised clustering based on methylation levels from both time points clearly showed that the methylation levels at the identified subset of CpG sites was markedly different between the first and second study time point in BTZ-treated cells (Figure 2, box A). At the same time, the methylation profiles from experimental controls mixed between the time points (Figure 2, box B), confirming that experimental conditions did not affect methylation levels at this subset of CpG sites.

**Figure 3:**
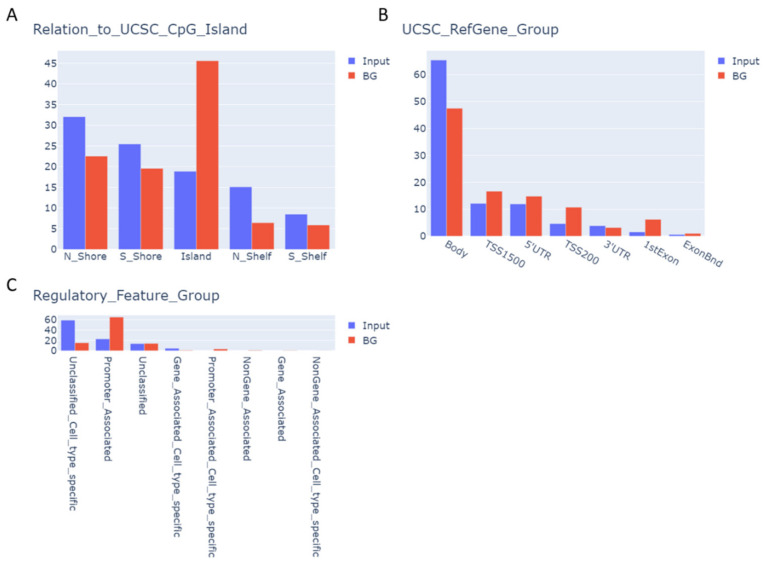
Distribution and relative enrichment of identified CpGs according to the functional regions of the genome, including the (**A**) CpG island, (**B**) gene region, and (**C**) regulatory features, as annotated in Illumina manifest. Input indicates set of 687 identified CpGs. BG—background.

We then mapped the identified CpGs to different genomic compartments, including CpG island regions and gene-specific regions. The CpG sites likely affected by BTZ treatment were significantly enriched at the N-Shelf (fold change (FC) = 1.8, *p* ≤ 0.05, chi-square goodness-of-fit test), and the gene body (FC = 1.4), and depleted at the CpG island (CGI; FC = 0.5), and at TSS200 (FC = 0.4). We also analyzed the distribution of these CpG sites in the regions predicted to regulate gene expression, referred to as regulatory features (informatically determined by ENCODE Consortium; Figure 3C), and found significant enrichment in “unclassified cell-type-specific regions” (FC = 2.2) and “non-gene-associated cell-type-specific regions” (FC = 11.8), simultaneous with the depletion in “promoter-associated regions” (FC = 0.4) and “promoter-associated-cell-type-specific regions” (FC = 2.1). This suggests that treatment of neuronal cells with BTZ induces methylation changes in specific regions of the genome, outside of the CGI and general promoters of the genes, but the regions harboring these changes are cell-type-specific.

### 3.4. BTZ-Induced Methylation Changes Affect Genes Involved in Neurogenesis and Neurotransmission

As shown in the above section, the most pronounced BTZ-induced methylation changes were not enriched within the well-known regulatory regions of the genome e.g., gene promoters or CGI. Recent studies, however, provide evidence for a causal relationship between methylation changes outside of these regions and gene transcription [23,24]. Therefore, to approximate the biological function of these lesions, we annotated CpGs with identified methylation changes to associated genes according to Illumina manifest, and used the GENE2FUNC function of FUMA GWAS (functional mapping and annotation of genome-wide association studies) [19] to perform gene set enrichment analysis (GSEA) based on the molecular signatures database (MSigDB) [25,26]. This analysis showed significant enrichment (FDR-corrected *p*-value < 0.05) of the genes harboring identified methylation changes in three ontology categories, including: GO biological processes (Supplementary Table S2), GO cellular components (Supplementary Table S3), and GO molecular function (Supplementary Table S4). Interestingly, the enriched terms in each of these ontology categories were similar, and associated with cell morphogenesis, neurogenesis, and neurotransmission. Specifically, top GO biological processes included “cell part morphogenesis” and “neuron differentiation”; however, most important in the context of mature neurons, a number of processes were associated with neurotransmission, including “regulation of membrane potential”, “synaptic signaling”, “ion transport”, and “regulation of cation channel activity”.

### 3.5. Specific Transcription Factor Binding Sites Are Affected by BTZ-Induced Methylation Changes

Next, we used the HOMER platform [20] to assess whether BTZ-related methylation changes affect the binding sites of specific transcription factors (TFs). This analysis showed that DNA motifs targeted by several TFs, including mainly EBF, PAX, DLX, LHX, and HNF family members, as well as En1, Nkx6.1, PRDM1, and Nanog transcription factors, were statistically significantly enriched in the regions harboring BTZ-induced methylation changes (*q*-value ≤ 0.05) (Supplementary Table S5). All of these TFs have previously been linked to neurogenesis or neuronal differentiation [27,28,29,30,31,32,33,34,35,36]; most interestingly, the top two identified TFs, PAX6 and EBF2, have been shown to be associated with peripheral neuropathy [37,38].

### 3.6. CpGs with BTZ-Induced Methylation Changes May Affect Chromatin Structure

We also used ATAC-Seq data available for differentiated LUHMES cells [39,40] to assess the enrichment of identified CpGs in open (i.e., active) chromatin regions. The identified CpGs were significantly depleted in open chromatin regions (FC = 0.1, chi-square goodness-of-fit test), which indicates that BTZ-induced methylation changes were localized in non-accessible chromatin regions. Therefore, it is plausible that changes in methylation at these CpG sites may affect the structure of chromatin, and change the accessibility of chromatin to, e.g., transcription factors.

### 3.7. BTZ-Induced Methylation Changes Identified in dLUHMES Cells Are Not Affected by BTZ Treatment in the SH-SY5Y Cell Line

**Figure 4:**
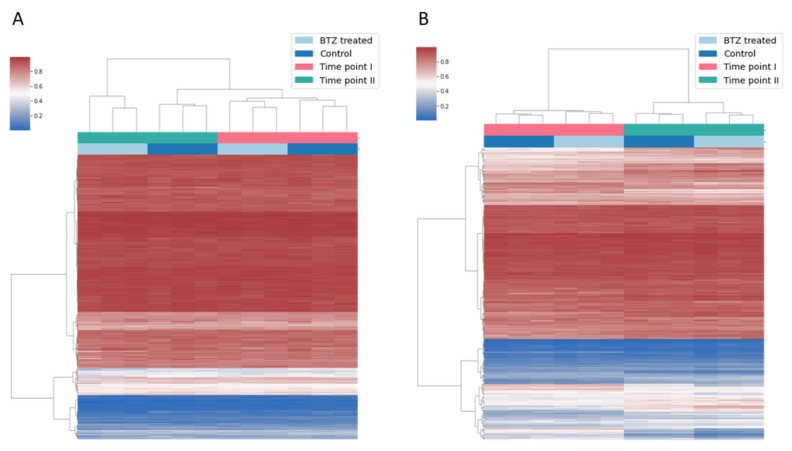
Heatmap illustrating unsupervised clustering of methylation changes identified to be affected by BTZ treatment in dLUHMES cells, based on data from our previous study of SH-SY5Y cells (**A**); heatmap illustrating methylation at the subset of CpGs previously identified to be affected by BTZ treatment in SH-SY5Y cells, based on data from the current study for dLUHMES cells (**B**).

We have recently published a study describing a key role of BTZ-induced methylation changes in the development of resistance to BTZ of the SH-SY5Y neuroblastoma cell line, subjected to treatment with this compound [13]. To investigate whether methylation changes induced by BTZ in dLUHMES cells are specific to these cells, we analyzed the methylation level of a subset of CpG sites that we identified to be affected by BTZ exposure, using the data from our previous study. As shown in Figure 4A, the methylation levels at the subset of the CpG sites identified in dLUHMES cells remained constant throughout the time points in our previous experiment. We also assessed the methylation levels at the subset of 4142 CpG sites that we previously reported to change in SH-SY5Y cells, which developed resistance to BTZ; interestingly, the methylation levels at these CpG sites did not change in dLUHMES cells during the experiment reported here (Figure 4B). These results indicate that the BTZ-induced methylation changes that we have identified in dLUHMES cells are most likely the effect of BTZ neurotoxicity.

## 4. Discussion

Recent studies have shown that DNA methylation of mature neurons changes in association with neuronal activity [41,42,43,44] and behavioral experience [45,46,47]. Thus, it was not surprising that, in our study, we observed methylome changes in differentiated LUHMES cells after BTZ treatment. Most of the changes we identified in our experiment were hypomethylation. In non-dividing cells, such as neurons, hypomethylation needs to be based on active demethylation. Currently, the most investigated active demethylation process is the oxidation of methylated cytosine (5mC) to 5-hydroxymethyl-cytosine (5hmC) [48], processed by TET (ten-eleven translocation) enzymes [49], and a number of studies have already demonstrated the ability of TETs to promote active demethylation and alter gene expression in response to neuronal activity [50,51,52,53,54,55].

Nevertheless, the largest methylation changes that we observed were gains in methylation. This process, in principle, requires the activity of de novo DNA methyltransferases (DNMTs). However, the post-mitotic neurons were shown to display expression of both de novo DNMT3a and maintenance DNMT1 methyltransferases, which perform promoter as well as gene body cytosine methylation [56]. Moreover, the critical role of the persistent expression of DNMT3a and DNMT1 in activity-dependent neuronal plasticity has been shown in vivo [57]. The coexistence of global hypomethylation and hypermethylation of the specific regions is one of the hallmarks of, for example, neoplastic transformation; therefore, it is not surprising that we observed both hyper and hypomethylation after BTZ treatment. However, it is interesting that the most significant methylation changes affected only a relatively small subset of the CpG sites that are specific to dLUHMES cells and appear to affect specific processes. These results suggest global deregulation of the methylation machinery under BTZ exposure, but despite high levels of randomness of the methylation changes, the significant methylation changes appear to have a specific direction. This speculation is also supported by the fact that TET proteins display little or no specificity for DNA sequences flanking the analyzed CpG site, suggesting that at least a subset of the identified methylation changes are random [58,59,60].

The CpG sites with the largest BTZ-related methylation change were enriched in regions with low CpG density (outside of CpG islands), such as gene bodies and the regions specific to the cell type. However, it is well established that methylation changes at single CpGs might not act on proximal, but distal genes [61]. More interestingly, the CpG sites with the largest methylation changes were depleted in euchromatin but enriched in heterochromatin, which suggests that methylation changes at these CpG sites may affect the structure of chromatin, and therefore change the accessibility of chromatin to, e.g., transcription factors.

The transcription factor binding sites that were enriched for the BTZ-induced methylation changes were EBF, PAX, DLX, LHX, and HNF family members, but also several other TFs, such as En1, Nkx6.1, PRDM1 and Nanog, all of which have previously been linked to neurogenesis or neuronal differentiation [27,28,29,30,31,32,33,34,35,36]. The top hit in this analysis was for PAX6, which has been shown to contribute to the painful sensation, referred to as mechanical allodynia, following applications of different chemotherapeutics treatments, including BTZ [37]. The second top transcription factor, EBF2, was shown in in vivo studies to play a key role in neuronal migration and nerve development [38]. Overall, this shows that methylation changes at the TF binding sites that we identified are likely to play a significant role in BTZ neurotoxicity, especially since the methylation levels at the subset of the CpG sites identified in this experiment did not change in SH-SY5Y cells, in which methylation changes induced by BTZ treatment lead to the development of proliferative phenotypes and treatment resistance [13].

Lastly, the functional analyses based on genes annotated as harboring BTZ-induced methylation changes showed that these genes are associated with neuronal physiology and neuron development. However, more important in the context of mature neurons, these genes were also linked to neurotransmission, including the regulation of membrane potential, synaptic signaling, ion transport, and regulation of cation channel activity, which is in line with previous in vivo studies showing associations between BTZ-induced disruption of the function of ion channels [62,63] and receptors involved in excitatory neurotransmission with neuropathic pain [64].

## 5. Conclusions

In conclusion, we show that changes in neural cell methylomes are induced by BTZ, and appear to be a part of the BTZ neurotoxicity that affects processes associated with the physiology of the neuron, including regulation of membrane potential, synaptic signaling, ion transport, and regulation of cation channel activity. The malfunction of the identified processes may contribute to the development of peripheral neuropathy in BTZ-treated patients; however, this hypothesis needs to be further investigated. It is also important to mention here that in our experiments, we were not able to rule out whether observed effects were a consequence of the direct influence of BTZ on DNA methylation machinery, or the indirect effect of proteasome inhibition. This needs to be investigated in the experiments following our publication.

## Data Availability

Methylation profiling data are deposited in NCBI Gene Expression Omnibus (GEO; https://www.ncbi.nlm.nih.gov/geo/; accessed on 14 June 2022) database under accession number GSE202792, and will be released upon the manuscript publication.

## Supplementary Materials

The following supporting information can be downloaded at: https://www.mdpi.com/article/10.3390/cancers14143402/s1, Table S1: CpGs with the most significant change of methylation across the time points in BTZ treated cells, Table S2: Significantly enriched GO terms in biological processes, Table S3: Significantly enriched GO terms in cellular components, Table S4: Significantly enriched GO terms in molecular function, Table S5: Analysis of transcription factor motif enrichment (HOMER).
